# Correlation of the Phase Angle with Muscle Ultrasound and Quality of Life in Obese Females

**DOI:** 10.1155/2022/7165126

**Published:** 2022-08-09

**Authors:** David Primo, Olatz Izaola, J. J. López Gómez, Daniel de Luis

**Affiliations:** ^1^Center of Investigation of Endocrinology and Clinical Nutrition, Medicine School, University of Valladolid, Valladolid, Spain; ^2^Dept Endocrinology and Nutrition Hospital Clinico Universitario, University of Valladolid, Valladolid, Spain

## Abstract

**Introduction:**

Phase angle (PhA) has been suggested to be an indicator of body cell mass and nutritional status. Clinically, the phase angle supposedly reflects body cell mass and cell membrane function, and the higher the phase angle, the better is the cell function. Muscle ultrasound (US) is an emerging nutritional assessment technique.

**Objective:**

The aim of this study was to investigate the usefulness and correlation of PhA with muscle US of quadriceps rectus femoris (QRF) in obese female subjects and the relationship with quality of life and physical performance. *Material and Methods*. In a total of healthy 50 obese female patients, anthropometric data by BIA, muscle mass by ultrasound at the QRF level, analytical determination, blood pressure, and quality of life were measured. Physical performance was assessed, too.

**Results:**

In total, 50 female obese patients were included with a mean age of 45.9 ± 2.4 years. The mean body mass index was 32.1 ± 1.6 kg/m^2^ with a mean weight of 83.5 ± 14.6 kg. Correlation analysis showed a positive correlation of PhA with all US parameters corrected by squared height (anteroposterior muscle thickness, circumference, cross-sectional area, and Echo-intensity). The correlation analysis of biochemical parameters with PhA showed a positive correlation with serum albumin and total protein levels. Physical activity and vitality scores of SF36 were correlated with PhA. Finally, PhA was positive correlated with physical performance, doing push-ups in 30 seconds (*r* =0.42; *p* =0.03) and doing squats in 30 seconds (*r* =0.54; *p* =0.02), without correlation with the time of 1.5 km walk.

**Conclusion:**

PhA was correlated with muscle area, muscle circumference, muscle echo intensity, serum protein, quality of life SF-36, and strength physical performance.

## 1. Introduction

The prevalence of obesity has increased considerably in recent years. It is estimated that more than 10% of adults are obese (11% in men and 15% in women) and up to 39% of adults are overweight (39% in men and 40% in women) [1]. Obesity is a risk factor for developing a lot of complications, such as arterial hypertension, dyslipidemia, cardiovascular events, osteoarthropathy, and cancer [2]. On the other hand, it is common to find a decrease in muscle mass in obese patients, which we call sarcopenia; the union of these two entities is called sarcopenic obesity [3]. Patients with these two situations have an even higher risk of metabolic disorders, a higher prevalence of cardiovascular diseases, high mortality rates, and lower physical activity [4]. Therefore, it is important to assess both fat mass and muscle mass in obese patients.

There is a lack of consensus on specific assessment muscle procedures as well as definition of muscle quality; a huge range of possibilities has been proposed, such as measurement of power and strength, ultrasonography, computed tomography, magnetic resonance imaging, dual-energy X-ray absorptiometry, and bioelectrical impedance (BIA) [5]. Bioelectrical impedance generates phase angle (PhA); this marker has increased attention for the assessment of nutrition status and it has been proposed as a variable useful for evaluating some structural aspects of muscle tissue [6, 7]. PhA is thought to be a representative of both proportion of body cell mass and water distribution (ratio extracellular water and total body water). PhA values might be altered in subjects with obesity [8] and it has been related to muscle strength [9], prognosis of some comorbidities [10], and quality of life [11].

In the last years, some investigations have reported the validity of ultrasound (US) to assess muscle quantity and quality status, by studying different muscle groups [12]. Moreover, these investigations have also used different measurements on different muscles, obtaining disparate results that are difficult to interpret and replicate, too. Nowadays, quadriceps rectus femoris (QRF) is a bipennate muscle, which has been investigated in different protocols of muscle evaluation with excellent results [13].

The aim of this study was to investigate the usefulness and correlation of PhA by BIA with muscle US of QRF in obese female subjects and the relationship of these data with quality of life and physical performance.

## 2. Material and Methods

The study recruited 50 obese female subjects without functional alterations that require a normal daily activity (Katz index 0) [14] who attended the consultations at our Hospital to assess their obesity (body mass index (BMI) ≥30 kg/m^2^). All 50 obese females agreed to participate in the study and all signed an informed consent for their inclusion before participating in the study. All patients finished the study without drop-outs. The scientific work was carried out in accordance with the Declaration of Helsinki and the protocol was approved by the Ethics Committee of our Centre (PI20/2062).

The inclusion criteria of the patients were the presence of obesity diagnosed with a BMI≥30 kg/m^2^, as well as normal activity in daily life. The exclusion criteria were the following: history of previous cardiovascular events, alcohol habit, active oncological process, and taking drugs during the 6 months prior to the study of drugs that influence lipid or glucose levels or who had followed a hypocaloric diet or a dietary supplement during this period.

During the protocol, the following data were collected: weight, height, body mass index (BMI), parameters by bioimpedance (BIA), waist circumference. and muscle parameters by ultrasound at the quadriceps rectus femoris. We measured blood pressure, physical performance with 3 physical tests (squats, push-ups, and a 1.5 km walk). and quality of life with the SF test-36, too. To determine the biochemical parameters, 5 ml of venous blood was aliquoted into tubes coated with ethylenediaminetetraacetic acid EDTA after an overnight fast of 10 hours. The following parameters were measured: albumin, total proteins, insulin, total cholesterol, LDL cholesterol, HDL cholesterol, triglycerides, and C-reactive protein.

### 2.1. Anthropometric Parameters, Bioimpedance, Muscle Ultrasound, and Blood Pressure

Height (cm) and waist circumference (cm) were measured with a nonelastic measuring tape (Omrom, LA, CA, USA). Body weight was determined with the subjects without clothing, using a digital scale (Omrom, LA, CA, USA). Using these parameters, the body mass index (BMI) (body weight (kg) divided by the square of the height (m)) was calculated.

The BIA was performed between 8:00 and 9 :15 hours, after an overnight fast and after a time of 15 minutes in the supine position. The BIA measured the geometrical components of impedance (*Z*); resistance (*R*), and the capacitance component (*X*). The PhA is derived for the next equation PhA = (*X*/*R*) × (180°/*π*). The BIA provided data regarding fat mass (FM), fat-free mass (FFM), skeletal muscle mass (SMM), appendicular muscle mass (aSMM), skeletal muscle mass index (SMI) as SMI divided by squared height, and appendicular muscle mass index (aSMMI) as SMMI divided by squared height [15] (EFG BIA 101 Anniversary, Akern, It). All these data are based on raw electrical data from BIA [14]. Sarcopenia has been excluded with a aSMMI >5.7 kg/m^2^ in females and a normal handgrip strength test [16].

Muscle ultrasound of the quadriceps rectus femoris (QRF) of the left and right lower extremities with a 10 to 12 MHz probe and a multifrequency linear matrix (Mindray Z60, Madrid, Spain) were performed in all subjects (patient in supine position). The probe was aligned perpendicular to the longitudinal and transverse axis of the QRF. The evaluation was performed without compression at the level of the lower third from the superior pole of the patella and the anterior superior iliac spine, measuring the anteroposterior muscle thickness, circumference, cross-sectional area, and the mean gray value (MGV) (Echo-intensity) (range 0-255). MGV was assessed considering the whole muscle longitudinal section as region of interest (ROI) in the same above-mentioned area of QRF. The gray scale spreads from 0 (black) to 255 (white). All US parameters were standardized dividing by the patient's height squared.

Mean systolic and diastolic blood pressures were calculated by averaging three consecutive measurements (Omrom, LA, CA, USA), after subjects sat for 10 minutes.

### 2.2. SF-36 Quality of Life Test and Assessment of Physical Performance

The SF-36 quality of life test was also performed with 11 items, which assesses 8 dimensions or health components. The participants were instructed to self-assess two strength tests and a 1500 meters walking. The subject would measure the following strength tests: upper body strength (maximum number of push-ups in 30 seconds), lower body strength (maximum number of squats in 30 seconds). They realized walking 1500 meters in the shortest time possible. The measurements were supervised and carried out by the patient together with one of the professional researchers, ensuring understanding, correct execution, and the way to record the results.

### 2.3. Biochemical Parameters

To evaluate the lipid profile, we determined the levels of albumin, total protein, total cholesterol, HDL cholesterol, and triglycerides using the COBAS INTEGRA 400 analyzer (Roche Diagnostic, Montreal, Canada). LDL cholesterol was calculated using the Friedewald formula (LDL cholesterol = total cholesterol-HDL cholesterol-triglycerides/5) [17]. Glucose levels were determined by an automated hexokinase oxidase method and insulin was measured by an electrochemiluminescence assay with the COBAS INTEGRA 400 analyzer (Roche Diagnostic, Montreal, Canada). To calculate insulin resistance, the Homeostasis model assessment (HOMA-IR) was calculated using these values (glucose × insulin/22.5) [18]. C-reactive protein (CRP) was measured by immunoturbimetry (Roche Diagnostics GmbH, Mannheim, Germany).

### 2.4. Statistical Analysis

Statistical analysis was performed with SPSS statistical software for Windows version 23.0 (SPSS Inc. Chicago, IL). *p* values below 0.05 were considered statistically significant. The sample size was determined to detect a significant correlation between PhA and muscle ultrasound parameters with 90% power and 5% significance (*n* =50). The Bonferroni test was applied for multiple tests to reduce type I error in the association analysis. Descriptive statistics for all variable values are presented as mean and standard deviation for continuous variables and as a percentage for categorical variables. The variables were analyzed with Student's *t* test (for the normal distribution variable) or the Kruskal-Wallis test (for the non-normal distribution variable). The chi-square test was used to assess the qualitative variables.

## 3. Results

In total, 50 female obese patients were included with a mean age of 45.9 ± 2.4 years. The mean body mass index was 32.1 ± 1.6 kg/m^2^ with a mean weight of 83.5 ± 14.6 kg.

Table 1 shows the classical anthropometric parameters and bioimpedance variables. No patient met the criteria for sarcopenia (aSSMI <5.7 kg/m^2^) in females. In Table 1, we report the different ultrasound parameters of the quadriceps rectus femoris, too.

Table 2 shows biochemical parameters, blood pressure.  Table 3 reports SF-36 results in eight dimensions. In the self-registration of 3 physical activities, we observed the following data; squats in 30 seconds (15.9 ± 2.9 times/30 sc), push-ups in 30 seconds (17.8 ± 5.5 times/30 sc), and the time in minutes needed to walk 1.5 km was 16.6 ± 3.3 minutes

Correlation analysis (Table 4) showed a positive correlation of PhA with all muscle ultrasound parameters corrected by squared height (anteroposterior muscle thickness, circumference, cross-sectional area, and the mean gray value (MGV) (Echo-intensity)). The correlation analysis of biochemical parameters with PhA showed a positive correlation of PhA with serum albumin and total protein levels. The remaining biochemical variables did not show a correlation with statistical association. The analysis of biochemical with ultrasound parameters did not show significative correlations (data not shown).

Table 5 shows the correlation analysis of PhA with SF-36 areas. Only physical activity and vitality scores were correlated with PhA. Neither biochemical parameter nor muscle ultrasound parameter correlated with the eight areas of SF36 (data not shown).

PhA was positive correlated with number of push-ups against a wall in 30 seconds (*r* =0.42; *p* =0.03) and number of squats in 30 seconds (*r* =0.54; *p* =0.02), without significant correlation with the time of 1.5 km walk. Neither ultrasound nor biochemical parameters showed a correlation with these physical performance parameters (data not shown).

## 4. Discussion

This is the first study evaluating the association between PhA and QRF ultrasound parameters with quality of life in obese females. Our study showed that PhA was correlated with ultrasound (US) parameters such as muscle area, muscle circumference, muscle echo intensity, and serum protein (albumin and total protein), too. Furthermore, PhA was correlated with quality of life and strength physical performance.

The results of the present study support the idea that BIA-derived PhA may be useful in the assessment of muscle status in obese females with an excellent correlation with QRF muscle ultrasound. As for muscle status, obesity shows an increased ratio of intramuscular and intermuscular fat infiltration and a decreased ratio of muscle mass to total body mass [10]. These situations are related with metabolic abnormalities [19], low strength [20], and decreased mobility [21]. In our study, obese females were not sarcopenic and had normal daily activity, and despite everything, PhA has been correlated with metabolic, clinical, and ultrasonography parameters, demonstrating its importance in the evaluation of this population. In other investigations, PhA has been related with muscle strength in different conditions [9], poor prognosis in chronic diseases [22], and impaired quality of life [11]. In this context, PhA might be regarded as an index of muscle quality [23], and the utility of this parameter on obesity patients has been addressed in few studies [24, 25]. The results of our sample of patients are interesting; it has been realized in grade I-II of obesity and in these stages, PhA is not influenced by obesity. PhA only tended to increase up to a BMI of 35 kg/m^2^.

On the other hand, some investigations using muscle ultrasound have found an association PhA and echogenicity of QFR in older subjects [26] and healthy adults [7]. Our study is the first to evaluate this association in obese females, showing a good correlation with parameters of cross-sectional area, circumference, thickness, and echo intensity of QFR. Muscle ultrasound has potential advantages; for example, Nakanishi et al. [27] reported that muscle ultrasound of QRF and biceps brachii is suitable for monitoring of muscle change in critically ill patients without the influence of fluid shift. This emerging field of ultrasound assessment of muscle mass needs for a standardization of measurement technique. Some guidelines have updated instructions for a large number of muscles (39 in total) [28] and different approaches for muscle ultrasound assessment were found that likely impact the values measured. It is necessary to standardize anatomical landmarks and measure points for all muscles/muscle groups. In our study, we have used the standardized point located at the distal third of the QRF, obtaining good results like other authors [13]. Despite this need for standardization of the measurement technique, muscle ultrasound has important advantages such as low economic cost, zero exposure to radiation, noninvasive technique, and a short period of time for exploration. And the good correlation with other radiation techniques has been demonstrated in different studies, for example the thickness of QRF with the aSMMI measured using a whole body DXA [29].

The relationship of body composition with quality of life and physical performance has been studied in patients with pulmonary fibrosis and using BIA [30]. In this study, PhA was correlated with SF-36 score and 6 minutes' walk test distance. These results were replicated in patients with psoriasis, in this case analyzing the quality of life with a specific test for dermatological pathology [31]. In these above-mentioned studies, PhA performed better to discriminate exercise capacity and quality of life than BMI or other body composition parameters. Muscle ultrasound was not used in both studies.

The PhA can be interpreted as a marker of cellular health [32] and of intra-and extracellular water distribution [33]; therefore, we can hypothesize that higher PhA values are related to better cell viability and therefore better vitality and physical activity by our obese subjects. Finally, our work shows a relationship between PhA and physical performance. Preceding studies have even related this parameter (PhA) with sports performance and competitive level in professional athletes [34, 35]. In our study, PhA was correlated with doing push-ups and squats as indicators of muscle mass and strength and no relationship was found with aerobic physical activity such as the 1.5 km walking test.

Some limitations of our study are to be acknowledged. First, the study is cross-sectional; correlations cannot differentiate cause and effect. Second, since ultrasound evaluation of skeletal muscle is not the gold standard, our results need to be taken with caution when evaluating only one muscle and by ultrasound (quadriceps femoris parameters). However, the use of an outpatient ultrasound compared to a computerized axial tomography of the third lumbar spine is much easier for clinical practice. Third, the study is carried out only in a small sample size females; therefore, we cannot generalize the data to the general population. Fourth, we use only a generic instrument to measure quality of life. Furthermore, the participation in this study was voluntary. This may have resulted in selection bias.

In conclusion, our study data showed that PhA was correlated with muscle area, muscle circumference, and muscle echo intensity by US and serum protein (albumin and total protein). PhA was correlated with quality of life and strength physical performance as doing squads and push-ups, too. Better understanding of simples' markers of muscle mass and strength is relevant since body composition in obese subjects is little evaluated. Since PhA is a direct derivative of the raw BIA parameters, it can be considered a good predictor of muscle mass, quality of life, and physical performance [36]. Further studies with a larger sample size and including men and women are necessary to improve the knowledge in this topic area.

## Figures and Tables

**Table 1:** Classical anthropometric, muscle ultrasound, and bioimpedance parameters.

Parameters	Mean ± SD
Weight (kg)	83.5 ± 14.6
BMI (kg/m^2^)	32.1 ± 1.6
Waist circumference	101.2 ± 10.6
Phase angle (°)	6.1 ± 0.7
Resistance Ohms	512.2 ± 68.7
Reactance Ohms	55.2 ± 9.9
Fat mass(kg)	33.2 ± 10.5
Fat-free mass (kg)	50.4 ± 5.6
SMM	25.1 ± 5.9
aSMM	19.9 ± 2.9
SMMI	9.7 ± 2.1
aSMMI	7.7 ± 0.9
Right muscle area QRF (cm^2^/m^2^)	1.61 ± 0.5
Left muscle area QRF (cm^2^/m^2^)	1.62 ± 0.6
Right circumference area QRF (cm/m^2^)	3.28 ± 0.5
Left circumference area QRF (cm/m^2^)	3.39 ± 0.6
Right thickness (cm/m^2^)	0.55 ± 0.1
Left thickness (cm/m^2^)	0.54 ± 0.2
Ecointensity (points/m^2^)	12.00 ± 5.8

BIA measured the geometrical components of impedance (Z), resistance (R), and the capacitance component (X). The BIA provided data regarding fat mass (FM), fat-free mass (FFM), skeletal muscle mass (SMM), appendicular muscle mass (aSMM), skeletal muscle mass index (SMMI) as SMMI divided by squared height, and appendicular muscle mass index (aSMMI) as SMI divided by squared height. QRF: quadriceps rectus femoris. Ultrasound parameters were standardized dividing by the patient's height squared; Y axis muscle thickness, circumference, cross-sectional area, and the mean gray value (MGV) (Echo-intensity).

**Table 2:** Biochemical parameters and blood pressure.

Parameters	Mean ± SD
Glucose (mg/dl)	92.6 ± 6.9
Total cholesterol (mg/dl)	199.1 ± 40.2
LDL cholesterol (mg/dl)	119.7 ± 23.6
HDL cholesterol (mg/dl)	53.1 ± 9.6
Tryglicerides (mg/dl)	118.4 ± 21.7
Insulin (UI/L)	14.6 ± 3.5
HOMA-IR	3.5 ± 1.4
CRP (mg/dl)	6.3 ± 1.2
Albumin (g/dl)	4.6 ± 0.4
Total protein (g/dl)	7.0 ± 0.5
Systolic blood pressure (mm/hg)	129.1 ± 12.5
Diastolic blood pressure (mm/hg)	78.0 ± 4.5

CRP: C-reactive protein; HOMA-IR: homeostasis model assessment.

**Table 3:** Quality of life SF36.

Parameters	Mean ± SD
General health	64.0 ± 20.9
Physical activity	83.7 ± 18.5
Physical Rol	84.5 ± 21.8
Emotional Rol	92.3 ± 21.8
Social function	95.5 ± 9.7
Pain	78.4 ± 25.4
Vitality	91.1 ± 13.4
Mental health	73.1 ± 13.5

**Table 4:** Correlation analysis between PhA and ultrasound and biochemical parameters.

Parameters Ultrasound	PhA	Biochemical parameters	PhA
Right muscle area QRF (cm^2^/m^2^)	*r* =0.57, *p* =0.001	Glucose (mg/dl)	*r* =0.11, *p* =0.21
Left muscle area QRF (cm^2^/m^2^)	*r* =0.56, *p* =0.001	Total cholesterol (mg/dl)	*r* =0.21, *p* =0.14
Right circumference area QRF (cm/m^2^)	*r* =0.42, *p* =0.002	LDL- cholesterol (mg/dl)	*r* =0.17, *p* =0.12
Left circumference area QRF (cm/m^2^)	*r* =0.48, *p* =0.001	HDL- cholesterol (mg/dl)	*r* =0.10, *p* =0.31
Right thickness Y axis (cm/m^2^)	*r* =0.69, *p* =0.001	Tryglicerides (mg/dl)	*r* =0.11, *p* =0.19
Left thickness Y axis (cm/m^2^)	*r* =0.62, *p* =0.002	Insulin (UI/L)	*r* =0.10, *p* =0.31
Ecointensity (points/m^2^)	*r* = -0.30, *p* =0.03	HOMA-IR	*r* =0.11, *p* =0.42
		CRP (mg/dl)	*r* =0.29, *p* =0.14
		Albumin (g/dl)	*r* =0.45, *p* =0.01
		Total protein (g/dl)	*r* =0.30, *p* =0.02

CRP: C-reactive protein; HOMA-IR: homeostasis model assessment.

**Table 5:** Correlation analysis between PhA and quality of life SF36.

Parameters	Areas of SF36
General health	*r* =0.17, *p* =0.21
Physical activity	*r* =0.12, *p* =0.52
Physical Rol	*r* =0.51, *p* =0.001
Emotional Rol	*r* =0.18, *p* =0.31
Social function	*r* =0.29, *p* =0.10
Pain	*r* =0.11, p =0.33
Vitality	*r* =0.32, *p* =0.03
Mental health	*r* =0.11, *p* =0.31

## Data Availability

All data generated or analyzed during this study are included in this article. Further enquiries can be directed to the corresponding author.
